# Improving the management of medical emergency team calls due to suspected infections: A before–after study^☆^

**DOI:** 10.1016/j.ccrj.2023.06.004

**Published:** 2023-08-09

**Authors:** Jeroen Ludikhuize, David Marshall, Misha Devchand, Steven Walker, Andrew Talman, Carmel Taylor, Tammie McIntyre, Jason Trubiano, Daryl Jones

**Affiliations:** a Austin Health, Department of Intensive Care Medicine in Heidelberg, Australia; b HagaZiekenhuis, Department of Intensive Care Medicine in the Hague, the Netherlands; c University Medical Center Amsterdam Location VuMC, Department of Acute Internal Medicine in Amsterdam, the Netherlands; d Austin Health, Department of Infectious Diseases in Heidelberg, Australia; e Austin Health, Department of Pharmacy in Heidelberg, Australia; f The University of Melbourne, Department of Surgery in Melbourne, Australia

**Keywords:** Medical emergency team, Infection, Sepsis, qSOFA, Implementation management protocol, Rapid response team, Clinical deterioration

## Abstract

**Objective:**

To introduce a management guideline for sepsis-related MET calls to increase lactate and blood culture acquisition, as well as prescription of appropriate antibiotics.

**Design:**

Prospective before (Jun–Aug 2018) and after (Oct–Dec 2018) study was designed.

**Setting:**

A public university linked hospital in Melbourne, Australia.

**Participants:**

Adult patients with MET calls related to sepsis/infection were included.

**Main outcome measures:**

The primary outcome measure was the proportion of MET calls during which both a blood culture and lactate level were ordered. Secondary outcomes included the frequency with which new antimicrobials were commenced by the MET, and the presence and class of administered antimicrobials.

**Results:**

There were 985 and 955 MET calls in the baseline and after periods, respectively. Patient features, MET triggers, limitations of treatment and disposition after the MET call were similar in both groups. Compliance with the acquisition of lactates (p = 0.101), respectively. There was a slight reduction in compliance with lactate acquisition in the after period (97% vs 99%; p = 0.06). In contrast, there was a significant increase in acquisition of blood cultures in the after period (69% vs 78%; p = 0.035).

**Conclusions:**

Introducing a sepsis management guideline and enhanced linkage with an AMS program increased blood culture acquisition and decreased broad spectrum antimicrobial use but didn't change in-hospital mortality.

## 1 Introduction

Sepsis is a major cause of morbidity and mortality in hospitalised patients.^1^^,^^2^ Important steps in the initial management of sepsis include the acquisition of blood cultures, measurement of serum lactate, and the early administration of appropriate antimicrobial therapy.^3^

The Medical Emergency Team (MET) is a team of clinicians who are called to review patients who have developed derangements in their vital signs.^4^ It is not surprising that approximately one-quarter of patients reviewed by the MET are thought to have an infection as the cause of their clinical deterioration.^5^ The importance of sepsis during MET calls has previously been emphasised, and the term sepsis rapid response teams has even been coined.^6^

Several elements of care in the early management of sepsis are associated with increased survival^7^ including timely commencement of antibiotics.^3^ However, inappropriate use of broad-spectrum antimicrobials is associated with the development of antimicrobial resistance.^8^ There is a paucity of literature exploring implementation of sepsis guidelines for patients who are in hospital wards.

The aim of our study was to evaluate the clinical management of infection-related MET calls comparing ‘usual care’ with care provided following implementation of structured guidelines. The aim was to investigate the effect of such guidelines on changes in the frequency of blood culture and serum lactate acquisitions and prescription of antibiotics during MET calls.

## 2 Methods

**Fig. 1:**
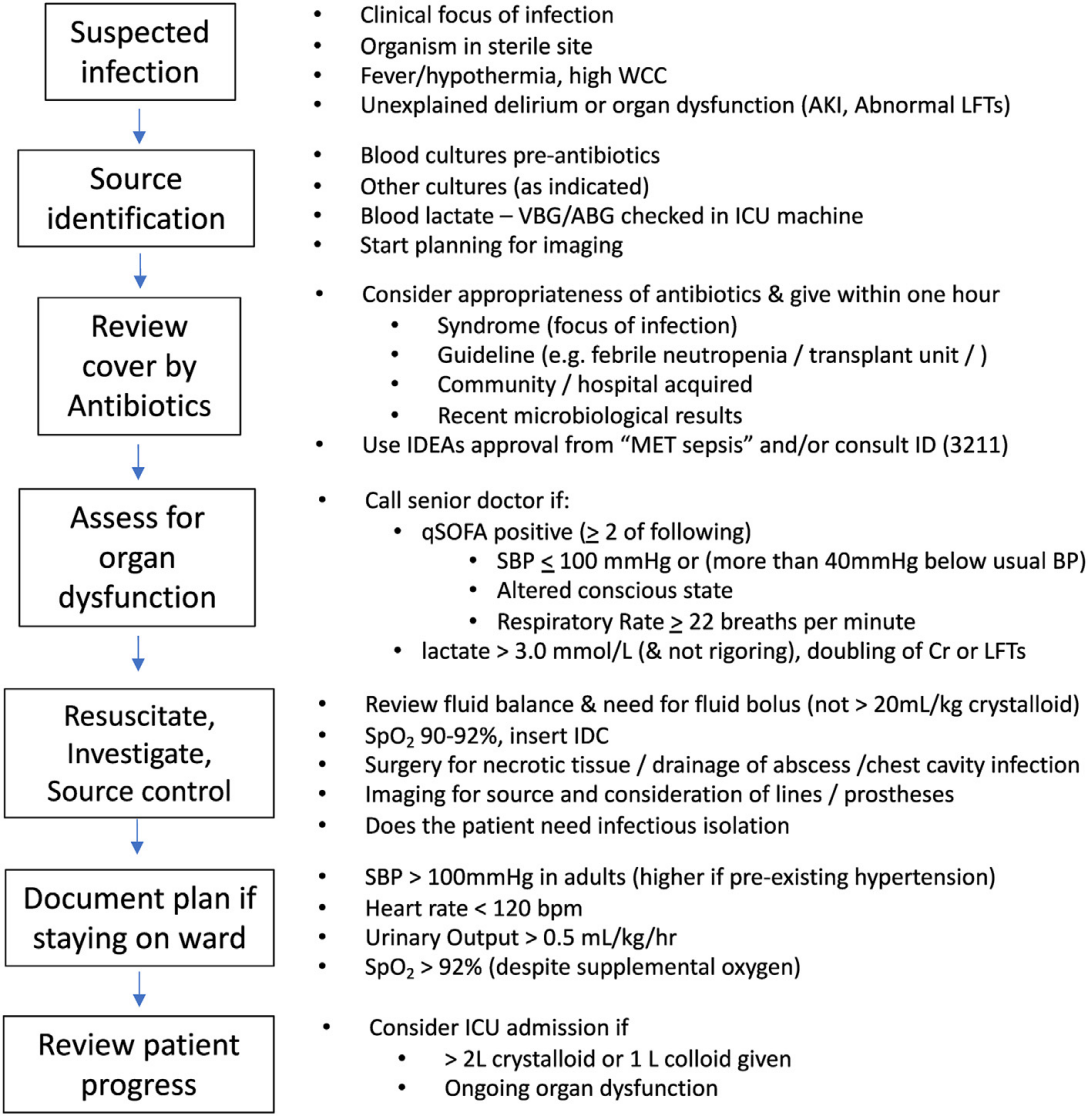
Flow diagram that appeared on MET trolley to guide the MET registrar in the initial management of MET calls due to presumed sepsis MET = Medical Emergency Team; WCC = white cell count; AKI = acute kidney injury; LFTs = liver function tests; VBG = venous blood gas; ABG = arterial blood gas; SOFA = sequential organ failure assessment; Cr = creatinine; IDC = in-dwelling catheter; bpm = beats per minute; kg = kilogramme; hr = hour; ICU = intensive care unit, IDEA approval = approval through the electronic antimicrobial decision support tool (see supplementary files).

The Austin hospital is a 450-bed University-affiliated hospital in Melbourne, Victoria, Australia. The MET was introduced into the hospital in October 2000 and has been described in detail previously.^9^ The MET is staffed 24/7 and is composed of an intensive care unit (ICU) registrar and senior nurses. Since April 2018, classification of septic MET calls was at clinician’s discretion based on the completion of a data field (“do you think the MET calls were due to sepsis?” [Yes/No]) in the electronic record completed by the attending MET registrar. As part of a before–after study, the baseline period (1st of June until August 31st, 2018) was compared with the period after the introduction of structured guidelines regarding sepsis management during MET calls (1st of October until the 31st of December 2018. A flow chart was available on the MET trolley, and an electronic antimicrobial stewardship process was developed. (Fig. 1).

For the patients with presumed sepsis, the health record was reviewed to identify the most extreme value for each of the vital signs in the 6 hr before and 6 hr after the activation of the MET call to evaluate whether and if the patient fulfilled the quick sequential organ failure assessment (qSOFA) criteria.^10^ In addition, we documented lactate, blood culture acquisitions, and antibiotic prescriptions during this timeframe. To enhance internal data validity, a random sample of 10% (n = 60) of patients was collected, and all data were entered separately and analysed using interobserver kappa coefficients.

The primary outcome measure was the proportion of MET calls, during which both a blood culture and a lactate level were ordered. Secondary outcomes included the frequency with which new antimicrobials were commenced by the MET and the presence and class of administered antimicrobials.

Additional information regarding implementation and statistical analysis can be found in the supplementary files section under the ‘methods’ heading.

## 3 Results

During the 6-months study period, there were 1940 MET calls, with 985 and 955 in the baseline and after periods (Table 1). Although there were no differences in the baseline demographics, there was a shorter hospital length of stay in the after period (10 [6–22]) than in the baseline period (14 [6.5–27]) (p < 0.001). To check for internal validity of the data, a data check was performed among 60 patients for the vital parameters comprising the qSOFA. There was a 12% disagreement for the respiratory rate and altered mental state (kappa 0.73) and a 5% disagreement for the recording of systolic blood pressure (kappa 0.89). This was deemed adequate to proceed.

**Table 1:** Medical Emergency Team calls with presumed infectious aetiology.

	Total	Before	After	p-value
Number (% of overall MET calls)	485 (24.5)	258 (26.2)	227 (23.8)	0.159
Unique patients (n, %)^a^	346	181	165	
Repeat MET calls (n, %)	123 (25)	71 (28)	52 (23)	0.244
Number of MET calls (med, IQR)	2 (1–2)	2 (1–3)	2 (1–2)	0.01
Age (median, IQR)^a^	69 (55–80)	70 (55–81)	68 (56–80)	0.491
Gender, female (n, %)^a^	145 (42)	73 (40)	72 (44)	0.586
qSOFA				
0	58 (12)	27 (11)	31 (14)	0.105
1	252 (52)	146 (57)	106 (47)	
≥2	166 (34)	83 (32)	86 (38)	
Missing	7 (1)	2 (1)	5 (2)	
MET immediate outcome				
Critical care	40 (10)	20 (8)	20 (9)	0.966
Operating room	1(0)	1 (0)	0	
Remained in ward	439 (91)	234 (91)	205 (90)	
Transferred	5 (1)	3 (1)	2 (1)	
Hospital outcome (n, %)^a^				
Home	205 (59)	106 (59)	99 (60)	0.955
Died	45 (13)	23 (13)	22 (13)	
Transfer	56 (16)	32 (18)	24 (15)	
Rehabilitation	14 (4)	7 (4)	7 (4)	
Other	26 (8)	13 (7)	13 (8)	
Mortality of qSOFA-positive MET call patients^a^	23 (20)	12 (22)	11 (18)	0.798
Compliance to protocol:				
Lactate taken (n, %)	476 (98)	256 (99)	220 (97)	0.06
Blood cultures taken (n, %)	351 (73)	177 (69)	174 (78)	0.035
Antibiotic prescriptions (n, %)				
Antibiotic pre MET	381 (79)	212 (83)	169 (75)	0.038
Antibiotic post MET	448 (92)	236 (92)	212 (93)	0.673
Antibiotic change	190 (40)	94 (37)	96 (43)	0.194
Overall compliance (n, %)	348 (72)	177 (69)	171 (75)	0.101

IQR: interquartile range; MET: Medical Emergency Team; qSOFA: quick sequential organ failure assessment

a The marked variables(s) have been displayed based on the unique number of patients to account for multiple MET calls.

MET calls secondary to sepsis were present in 24.5% of MET calls overall; 26.2% in the baseline period; and 23.8% in the after period, respectively (p = 0.159). There was less physiological derangement in relation to oxygen saturations, respiratory rate, and heart rate in the after period. However, more patients in the after period had an impaired conscious state, and the measured lactate was higher (see supplementary tables 1 and 2). Despite these differences, the proportion of patients with a qSOFA of ≥2 in both time periods was similar (p = 0.105) (suppl. Table 2).

Compliance with the acquisition of lactate and blood cultures was 69% in the before and 75% in the after periods (p = 0.101), respectively. There was a slight reduction in compliance with lactate acquisition in the after period (97% vs 99%; p = 0.06). In contrast, there was a significant increase in acquisition of blood cultures in the after period (69% vs 78%; p = 0.035). After the MET call, most patients were on antibiotics (96 versus 92%, p = 0.67) with reduced prescriptions for broad-spectrum antibiotics (supplementary table 3).

## 4 Discussion

The diagnosis of sepsis is a frequent occurrence in the hospital and is encountered by the MET frequently. Our study was developed to enhance sepsis care in ward patients by a structured approach during these calls. Lactate acquisition was high at the baseline, and more than two-thirds of patients had blood cultures taken during the baseline period. Therefore, no significant difference in overall compliance could be identified. There was an overall trend towards reduced broad-spectrum antibiotic use in the after period. To our knowledge, this is the largest study exploring issues around sepsis during MET calls.

Our study has several limitations including before and after design, seasonal variation in MET activity and case-mix, presence of delay of MET activation, and differences of the severity of vital sign derangements between the two cohorts. Moreover, more patients were already on antibiotics at the time of the MET call in the baseline period. These factors may have affected both the frequency of presumed infections and outcomes, although the latter is not a (primary) outcome.

Our exploratory study indicates that with a structured approach, changes can be made in sepsis management. Our study was not powered to assess for a change in patient outcomes. However, we believe that the guideline developed and having this at the point of care are readily generalisable to other institutions.

Similar structured approaches could potentially be used for other serious conditions (arrhythmias, end of life/terminal illness, etc.) encountered during MET calls. Future studies will need to focus on bringing decision support to increase overall compliance and escalation of care in case of refractory symptoms to more senior ICU doctors and analyse it's influence on relevant patient outcomes.

## 5 Conclusions

Our study shows that one quarter of MET calls were sepsis-related. We successfully developed a streamlined and structured guideline for managing septic MET calls, along with targeted education to members of the MET and the use of an online antimicrobial stewardship program. This approach resulted in enhanced blood culture acquisition and a reduction in broad-spectrum antibiotic usage.

### CRediT authorship contribution

**Jeroen Ludikhuize**: Conceptualization, Methodology, Formal analysis, Data Curation, Writing - Initial Draft, Supervision. **David Marshall**: Investigation. **Misha Devchand**: Investigation, Writing - Initial Draft. **Steven Walker**: Project Administration. **Andrew Talman**: Validation, Investigation. **Carmel Taylor**: Project Administration. **Tammie McIntyre**: Project Administration. **Jason Trubiano**: Methodology, Writing – Review & Editing. **Daryl Jones**: Conceptualization, Methodology, Writing – Review & Editing, Supervision.
