# Supplementary material for: Improving the management of medical emergency team calls due to suspected infections: A before–after study
Source: Crit Care Resusc. 2023 Aug 9;25(3):136–9. doi: 10.1016/j.ccrj.2023.06.004 (PMC10581256; doi:10.1016/j.ccrj.2023.06.004)
Supplement: Multimedia component 1 [file mmc1.doc]

**Online supplement.**

**Improving the management of medical emergency team calls due to suspected infections. A before after study.**

Authors:

J. Ludikhuize ^1,2,3^, D. Marshall ^1^, M. Devchand ^4,5^, S. Walker ^4^, A. Talman ^1^, C. Taylor ^1^, T. McIntyre ^1^, J. Trubiano ^4^ and D. Jones ^1,6^

^1^ Austin Health, department of Intensive Care medicine in Heidelberg, Australia

^2^ HagaZiekenhuis, department of Intensive Care medicine in The Hague, The Netherlands

^3^ University Medical Center Amsterdam location VuMC, department of acute internal medicine in Amsterdam, The Netherlands

^4^ Austin Health, department of Infectious Diseases in Heidelberg, Australia

^5^ Austin Health, department of Pharmacy in Heidelberg, Australia

^6^ The University of Melbourne, department of Surgery in Melbourne, Australia

Corresponding author:

Jeroen Ludikhuize, [j.ludikhuize@hagaziekenhuis.nl](mailto:j.ludikhuize@hagaziekenhuis.nl)

**Supplementary information regarding methods used including detailed information in relation to the implementation.**

Study design and study periods

This was a before-and study aimed at improving compliance with agreed steps in the early management of sepsis. The baseline period was conducted between Jun 1^st^ – Aug 31^st^ (winter) 2018. During Sep there was extensive education of the registrars and nurses involved in the MET response. The period Oct 1^st^ to Dec 31^st^ (Spring and summer) 2018 was considered the “after” period.

Details of education and quality improvement processes

A one-page flow diagram (figure 1) outlining important steps in the early management of sepsis was developed by senior doctors. During September, the wording and order of the steps of the guideline were modified slightly based on feedback and input from ICU registrars involved in the MET.

The lead investigators and two senior ICU nurses involved in the MET provided education in-services to the MET nurses and registrars during September. This was initiated by a planned meeting including the presentation of the flow diagram and other logistics involved. Post-meeting, regular face-to-face meetings with all registrars involved in the MET was performed including email communication and reminders.

The single page summarising the flow diagram was positioned on the MET trolley and a hospital-wide guideline was approved and made available organisation wide. The flow diagram also provided decision support at the point of care to link with the hospital antimicrobial stewardship (AMS) program. The AMS program has an electronic decision support approval system (IDEA^3^S) (1), electronic medical record and clinician support has been established at Austin Health and details previously published (2). IDEA^3^S enabled clinicians to obtain 24-hour electronic approvals for antimicrobial agents, using a specific indication of “MET sepsis”. The contact details of the infectious disease registrar on-call were also provided for further advice. All MET sepsis approvals were reviewed the following day by the AMS team.

Details of data collected

We collected details on the number of MET calls in each period and patient demographics. We also recorded whether the admitting unit was medical or surgical, the admission was emergency in nature, and whether the patient admission occurred after hours (18:01 – 07:59). Details of the MET call included the time of activation, the reason for the call, and whether there were treatment limitations before or after the call. We also recorded the immediate outcome of the MET call, as well as the hospital length of stay, and disposition at the time of hospital discharge.

Classification of septic MET calls was at clinician discretion based on the completion of the data field in the electronic entry completed by the attending MET registrar at the end of the MET call. For these patients the health record was reviewed to identify the most extreme value for each of the vital signs in the 6hr before and 6hr after the activation of the MET. In addition, we also documented whether the patient fulfilled quick Serial Organ Failure Assessment criteria (SBP < 100mmHg, altered conscious state, RR $\geq$ 22 breaths/min) as previously published (3). Further, we documented whether a lactate and blood cultures were taken, and the value of the lactate. We also recorded whether the patient was on systemic antibiotics before the MET (including peri-operative prophylactic antibiotics) or therapeutic antibiotics (to treat presumed infection), and whether the antibiotics were modified or commenced after the MET.

To enhance internal data validity, a random sample of 10% (n=60) patients were collected and all data entered separately. Interobserver kappa coefficients were calculated for the observed respiratory rate, systolic blood pressure, altered mental state and the calculated qSOFA score.

In a randomly selected cohort of septic patients’ classes of antimicrobial prescribing was evaluated by two independent pharmacists. This evaluation included existing antimicrobials as well as antimicrobials commenced during the MET call.

**Supplementary table 1. Patient demographics, details of MET calls, immediate and in-hospital outcomes for MET calls during entire study period.**

|  | **All patients** | **Before**  **(Jun-Aug)** | **After**  **(Oct-Dec)** | **p-value** |
| --- | --- | --- | --- | --- |
| Number of MET calls | 1940 | 985 | 955 |  |
| Unique patients (n, %) *^1^ | 1156 | 581 | 575 |  |
| Gender, female (n, %) *^1^ | 534 (46) | 261 (45) | 273 (47) | 0.409 |
| Age (median, IQR) years *^1^ | 72 (57 – 82) | 72 (55 – 83) | 71 (58 – 82) | 0.990 |
| Unit of MET call, Medical (n, %) | 1272 (66) | 655 (66) | 617 (65) | 0.496 |
| Emergency admission (n, %) *^1^ | 783 (68) | 384 (66) | 399 (69) | 0.721 |
| MET activation, after hours (n, %) | 1324 (68) | 686 (70) | 638 (67) | 0.188 |
| Patients with repeat MET calls (n, %) | 368 (30) | 184 (29) | 184 (31) | 0.709 |
| Reason for MET call (n, %) |  |  |  |  |
| Breathing difficulty | 34 (2) | 19 (2) | 15 (2) | 0.851 |
| Change in GCS | 141 (7) | 79 (8) | 62 (7) |  |
| Chest pain | 30 (2) | 10 (1) | 20 (2) |  |
| Hight heart rate | 522 (27) | 274 (28) | 248 (26) |  |
| High respiratory rate | 366 (19) | 186 (19) | 180 (19) |  |
| Low blood pressure | 391 (20) | 194 (20) | 197 (21) |  |
| Low heart rate | 26 (1) | 14 (1) | 12 (1) |  |
| Low oxygen saturation | 209 (11) | 100 (10) | 109 (11) |  |
| Low respiratory rate | 11 (1) | 6 (1) | 5 (1) |  |
| Seizures | 30 (2) | 15 (2) | 15 (2) |  |
| Low urine output | 3 (0) | 1 (0) | 2 (0) |  |
| Noisy breathing/Stridor | 6 (0) | 3 (0) | 3 (0) |  |
| Obstructed airway | 2 (0) | 1 (0) | 1 (0) |  |
| Patient bleeding | 13 (1) | 5 (1) | 8 (1) |  |
| Problems with tracheostomy | 1 (0) | 0 | 1 (1) |  |
| Severe/Uncontrolled pain | 41 (2) | 19 (2) | 22 (2) |  |
| Worried | 114 (6) | 59 (6) | 55 (6) |  |
| Treatment limitation present at admission (n, %) *^1^ |  |  |  |  |
| Full Care | 791 (68) | 387 (67) | 404 (70) | 0.370 |
| Not for respond Blue, for MET | 352 (30) | 188 (32) | 164 (29) |  |
| Not for respond Blue and MET | 13 (1) | 6 (1) | 7 (1) |  |
| Treatment limitation present at discharge (n, %) *^1^ |  |  |  |  |
| Full Care | 745 (64) | 360 (62) | 385 (67) | 0.132 |
| Not for respond Blue, for MET | 356 (31) | 188 (32) | 168 (29) |  |
| Not for respond Blue and MET | 55 (5) | 33 (6) | 22 (4) |  |
| Direct outcome after MET call (n, %) |  |  |  |  |
| Remained on current ward | 1741 (90) | 876 (89) | 865 (91) | 0.474 |
| Critical Care transfer | 129 (7) | 70 (7) | 59 (6) |  |
| Operating room | 13 (1) | 5 (1) | 8 (1) |  |
| Died during MET | 4 (0) | 2 (0) | 2 (0) |  |
| Other | 53 (3) | 32 (3) | 21 (2) |  |
| Discharge destination *^1^ |  |  |  |  |
| Home | 672 (58) | 324 (56) | 348 (61) | 0.311 |
| Transfer care facility | 261 (23) | 135 (23) | 126 (22) |  |
| Died in hospital | 127 (11) | 72 (12) | 55 (10) |  |
| Other | 96 (8) | 50 (9) | 46 (8) |  |
| LOHS hospital, days (median, IQR) *^1^ | 10 (5 – 20) | 10 (5 – 20) | 10 (5 – 19) | 0.843 |

*^1^ The marked parameter(s) have been displayed based on the unique number of patients therefore accounting for multiple MET calls.

MET = medical emergency team; GCS = Glasgow Coma Score; LOHS = length of hospital stay; IQR = inter-quartile range

**Supplementary table 2. Patient demographics and outcomes for MET calls with presumed infective aetiology.**

|  | **Total** | **Before** | **After** | **p-value** |
| --- | --- | --- | --- | --- |
| Number (% of overall MET calls) | 485 (24.5) | 258 (26.2) | 227 (23.8) | 0.159 |
| Unique patients (n, %) | 346 | 181 | 165 |  |
| Repeat MET calls (n, %) | 123 (25) | 71 (28) | 52 (23) | 0.244 |
| Number MET calls (med, IQR) *^1^ | 2 (1 - 2) | 2 (1 - 3) | 2 (1 - 2) | <0.001 |
| Age (median, IQR) *^2^ | 69 (55 – 80) | 70 (55 – 81) | 68 (56 – 80) | 0.491 |
| Gender, female (n, %) *^2^ | 145 (42) | 73 (40) | 72 (44) | 0.586 |
| MET activation after hours (n,%) | 339 (69.9) | 183 (70.9) | 156 (68.7) | 0.597 |
| Unit of MET call, Medical (n, %) | 320 (66) | 174 (67.4) | 146 (64.3) | 0.268 |
| Reason for MET call (n, %) |  |  |  |  |
| Breathing difficulty | 8 (2) | 4 (2) | 4 (2) | 0.881 |
| Change in GCS | 9 (2) | 4 (2) | 4 (2) |  |
| Chest pain | 1 (0) | 0 | 1 (0) |  |
| Hight heart rate | 171 (35) | 93 (36) | 78 (34) |  |
| High respiratory rate | 110 (23) | 62 (24) | 48 (21) |  |
| Low blood pressure | 107 (22) | 53 (21) | 54 (24) |  |
| Low heart rate | 1 (0) | 1 (0) | 0 |  |
| Low oxygen saturation | 59 (12) | 29 (11) | 30 (13) |  |
| Low respiratory rate | 2 (0) | 1 (0) | 1 (0) |  |
| Seizures | 1 (0) | 1 (0) | 0 |  |
| Severe/Uncontrolled pain | 1 (0) | 1 (0) | 0 |  |
| Worried | 15 (3) | 9 (4) | 6 (3) |  |
| Vital sign (Median, IQR) |  |  |  |  |
| Lowest SpO_2_ | 94 (91 - 96) | 94 (90 - 95) | 95 (92 - 96) | <0.001 |
| Highest resp rate | 26 (21 - 32) | 27 (22 - 32) | 24 (20 - 30) | <0.001 |
| Lowest resp rate | 18 (16 - 21) | 18 (16 - 20) | 18 (17 - 22) | 0.138 |
| Highest heart rate | 115 (98 - 131) | 120 (100 - 134) | 109 (97 - 127) | 0.001 |
| Lowest systolic BP | 106 (91 - 122) | 107 (90 - 124) | 105 (92 - 121) | 0.967 |
| Lowest diastolic BP | 61 (50 - 70) | 61 (50 - 70) | 61 (50 - 70) | 0.813 |
| Highest temperature | 38 (37 – 38.7) | 38.1 (37.1 – 38.7) | 37.9 (36.9 – 38.7) | 0.211 |
| Lowest temperature | 36.7 (36.2 – 37.3) | 36.7 (36.1 – 37.3) | 36.8 (36.2 – 37.3) | 0.561 |
| Highest lactate | 1.4 (0 – 2.2) | 1.3 (0 – 2.1) | 1.5 (0.7 – 2.4) | 0.036 |
| GCS <15 (n, %) | 96 (20) | 39 (15) | 57 (26) | 0.005 |
| qSOFA |  |  |  |  |
| 0 | 58 (12) | 27 (11) | 31 (14) | 0.105 |
| 1 | 252 (52) | 146 (57) | 106 (47) |  |
| ≥2 | 166 (34) | 83 (32) | 86 (38) |  |
| Missing | 7 (1) | 2 (1) | 5 (2) |  |
| MET goals before |  |  |  |  |
| Full care | 353 (73) | 182 (71) | 171 (75) | 0.474 |
| Not for blue, for MET | 128 (26) | 74 (29) | 54 (24) |  |
| Not for blue/MET | 4 (1) | 2 (1) | 2 (1) |  |
| MET goals after |  |  |  |  |
| Full care | 329 (68) | 173 (67) | 156 (69) | 0.862 |
| Not for blue, for MET | 139 (29) | 75 (29) | 64 (28) |  |
| Not for blue/MET | 17 (4) | 10 (4) | 7 (3) |  |
| MET immediate outcome |  |  |  |  |
| Critical Care | 40 (10) | 20 (8) | 20 (9) | 0.966 |
| Operating room | 1(0) | 1 (0) | 0 |  |
| Remained on ward | 439 (91) | 234 (91) | 205 (90) |  |
| Transferred | 5 (1) | 3 (1) | 2 (1) |  |
| Hospital outcome (n, %) *^2^ |  |  |  |  |
| Home | 205 (59) | 106 (59) | 99 (60) | 0.955 |
| Died | 45 (13) | 23 (13) | 22 (13) |  |
| Transfer | 56 (16) | 32 (18) | 24 (15) |  |
| Rehabilitation | 14 (4) | 7 (4) | 7 (4) |  |
| Other | 26 (8) | 13 (7) | 13 (8) |  |
| Mortality of q-SOFA positive MET call patients *^2^ | 23 (20) | 12 (22) | 11 (18) | 0.798 |
| LOHS before MET Call (days, IQR) | 4 (1 – 13) | 4 (1 – 14) | 3 (1 – 12) | 0.132 |
| LOHS after MET Call (days, IQR) | 9 (4 – 20) | 10 (5 – 23) | 9 (4 – 16) | 0.028 |
| LOHS (days, IQR) *^2^ | 13 (6 – 26) | 12 (7 – 27) | 13 (6 – 26) | 0.827 |

*^1^ Number of calls in case of repeat MET calls

*^2^ The marked parameter(s) have been displayed based on the unique number of patients therefore accounting for multiple MET calls.

MET = medical emergency team; GCS = Glasgow Coma Score; LOHS = length of hospital stay; IQR = inter-quartile range; SOFA = sequential organ failure assessment

**Supplementary table 3. Antibiotic prescription and classes for MET reviews with presumed infectious aetiology.**

|  | **All** | **Before** | **After** |  |
| --- | --- | --- | --- | --- |
| Patients (n, %) | 260 | 158 | 102 | - |
| Unique patients (n, %) *^1^ | 214 (82) | 122 (77) | 92 (90) | 0.040 |
| Age (med, IQR) *^1^ | 67 (52 – 78) | 69 (56 – 81) | 64 (47 – 75) | 0.009 |
| Gender, female (n, %) *^1^ | 85 (40) | 42 (34) | 43 (47) | 0.056 |
| Unit of MET call, Medical (n, %) | 151 (58) | 86 (54) | 65 (64) | 0.157 |
| On antibiotics before MET (n, %) | 151 (58) | 109 (69) | 42 (41) | <0.001 |
| Antibiotic change during MET | 53 (20) | 47 (30) | 6 (6) | <0.001 |
| Antibiotics started at MET | 79 (30) | 33 (21) | 46 (45) | <0.001 |
| Antimicrobial 1 (n, %) *^2^ |  |  |  |  |
| Piperacillin-Tazobactam | 40 (32) | 26 (35) | 14 (26) |  |
| Ceftriaxone | 33 (26) | 20 (27) | 13 (25) |  |
| Amoxicillin-clavulanic acid | 3 (3) | 3 (4) | 0 |  |
| Gentamycin | 9 (7) | 5 (7) | 4 (8) |  |
| Meropenem | 7 (6) | 3 (4) | 4 (8) |  |
| Vancomycin | 8 (6) | 4 (5) | 4 (8) |  |
| Cefepime | 5 (4) | 0 | 5 (9) |  |
| Azithromycin | 5 (4) | 1 (1) | 4 (8) |  |
| Antimicrobial 2 (n, %) *^2^ |  |  |  |  |
| Flucloxacillin | 4 (12) | 3 (14) | 1 (8) |  |
| Metronidazole | 5 (15) | 2 (10) | 3 (23) |  |
| Gentamycin | 4 (12) | 3 (14) | 1 (8) |  |
| Teicoplanin | 3 (9) | 3 (14) | 0 |  |
| Vancomycin | 2 (6) | 2 (10) | 0 |  |
| Azithromycin | 5 (15) | 3 (14) | 2 (15) |  |

*^1^ Number of calls in case of repeat MET calls

***^2^** Represents top 5 of antibiotics prescribed at the time of the MET call from each study period therefore total number of listed antibiotics exceeds 5.

**References**

1. Grayson ML, Melvani S, Kirsa SW, Cheung S, Garrett MK, Korman AM, et al. Impact of an electronic antibiotic advice and approval system on antibiotic prescribing in an Australian teaching hospital. Med J Aust. 2004 May 3;180(9):455–8.

2. Devchand M, Nolen A, Stewardson AJ, Warrillow SJ, Garrett K, Trubiano JA. Long-term outcomes of an electronic medical record (EMR)–integrated antimicrobial stewardship (AMS) intensive care unit (ICU) ward round. Infect Control Hosp Epidemiol. 2021;1–3.

3. Singer M, Deutschman CS, Seymour CW, Shankar-Hari M, Annane D, Bauer M, et al. The Third International Consensus Definitions for Sepsis and Septic Shock (Sepsis-3). JAMA. 2016 Feb 23;315(8):801-10.
